# Maternal Dietary Protein Patterns During Pregnancy and the Risk of Infant Eczema: A Cohort Study

**DOI:** 10.3389/fnut.2021.608972

**Published:** 2021-06-02

**Authors:** Jingjing Zeng, Weijia Wu, Nu Tang, Yajun Chen, Jin Jing, Li Cai

**Affiliations:** ^1^Department of Maternal and Child Health, School of Public Health, Sun Yat-sen University, Guangzhou, China; ^2^Department of Scientific Research, Hainan Women and Children's Medical Center, Haikou, China; ^3^Guangdong Key Laboratory of Nutrition, Diet and Health, Guangzhou, China

**Keywords:** pregnancy, protein, dietary pattern, food sources, infant eczema

## Abstract

**Background:** Previous studies have suggested that maternal dietary protein was associated with allergic diseases in offspring, but few studies have evaluated the influence of dietary protein patterns. This study aimed to explore the prospective association between maternal dietary protein patterns during pregnancy and the risk of infant eczema.

**Methods:** A total of 713 mother-child pairs from a prospective cohort in Guangzhou, China were recruited. Maternal dietary protein was estimated using a validated face-to-face food frequency questionnaire at 20–28 weeks' gestation from 2017 to 2018. Dietary protein patterns were calculated based on the sources of protein. The data of infant eczema was assessed at 6 months of age using the symptom questionnaire of eczema. Logistic regression was carried out to examine the associations between maternal dietary protein patterns and infant eczema.

**Results:** The cumulative incidence of infant eczema at 6 months of age was 51.19%. Mothers of infants with eczema consumed more protein from poultry source during pregnancy than mothers of infants without eczema, while no statistical differences were observed in maternal intakes of protein from cereals and tubers, vegetables, fruits, red meat, fish and seafood, eggs, dairy, soybean, and nuts and seeds. Four dietary protein patterns were identified and termed poultry, plant, dairy and eggs, and red meat and fish. The cumulative incidence of eczema was 61.2, 45.8, 48.0, 51.4% for these four patterns, respectively. Compared to the poultry dietary pattern, the plant pattern and the dairy and eggs pattern were associated with a reduced risk of infant eczema, and the adjusted odds ratios (95% confidence interval) were 0.572 (0.330–0.992), 0.478 (0.274–0.837), respectively. No such association was observed for the red meat and fish dietary protein pattern.

**Conclusion:** This is the first study that focused on the association between maternal dietary protein during pregnancy from a whole-diet perspective and infant eczema. Compared with the poultry dietary protein pattern, the maternal plant pattern and the dairy and eggs pattern during pregnancy were associated with a reduced risk of infant eczema.

## Introduction

Eczema is one of the most frequent chronic inflammatory skin diseases and it most often occurs in early infancy (1). Infants with eczema tend to have an increased risk for food allergy, wheezing, asthma, allergic rhinitis, and subsequent psychosocial and behavioral issues (2–5). The pathogenesis of infant eczema is not yet well-understood but believed to be influenced by genetic factors and environmental exposures (1). In the past 30 years, the prevalence of eczema has increased globally (6). Environmental factors are likely to play an increasingly important role. According to the DOHaD (Developmental Origins of Health and Disease) hypothesis (7), non-inheritable exposures *in utero* can alter offspring programming of immune function and developing of allergic diseases (8, 9). Maternal diet during pregnancy can provide nutrients to fetal development, and also may impact fetal immune responses (10).

The impaired epidermal barrier is one of the hallmarks of eczema (11), enhancing penetration of the skin by allergen and microbes (12). Maternal dietary protein, as an important nutrient and major allergens, can influence fetal growth and development, such as epidermal structure and function as well as immune system (13, 14). The association between maternal dietary proteins and offspring allergic diseases has long been discussed. Previous studies suggested that maternal intake of some food allergens (e.g., milk and peanut) during pregnancy may reduce the risk of food allergy (15, 16). There is a strong association between eczema and food allergy, with approximately one-third of all patients with severe eczema documented for food allergy (17). Maternal ingestion of wheat during mid-pregnancy was associated with decreased childhood eczema (18), and higher intake of dairy products during pregnancy was associated with reduced risk of infant eczema and asthma (19). As nutritional support for fetal development, proteins are generally digested into amino acids and transport to the fetus through the placenta (20). Nevertheless, the intact major food allergens (from milk, eggs, fish, fruits, nuts, and wheat) were detected in maternal amniotic fluid during pregnancy (21), and experimental evidence *in vitro* prove the view of transplacental allergens transfer (22–24). Early-life exposure of nutritive allergens could modulate fetal immune development and affect immune responses to allergens exposed after birth (25).

Since the diet consists of various foods and complex nutrients, dietary pattern analysis would parallel more closely the actual situation (26). Dietary patterns can reflect the individual diet habit over a period of time. Since the foundations of contemporary dietary guidelines are based on dietary patterns (27) and dietary patterns of protein intake may influence the willingness of people to modify their dietary behavior (28), it is more practical to focus on proteins from the perspective of dietary patterns. Individual protein intake from different food sources is regarded as a whole and evaluated comprehensively. Therefore, it is practical and instructive to investigate people's dietary intake to determine their dietary patterns. To date, there is no available evidence for maternal dietary protein patterns and infant eczema. Therefore, the aim of current study is to investigate the association between maternal dietary protein patterns during pregnancy and the risk of infant eczema.

## Materials and Methods

### Study Design and Population

Data was drawn from an on-going prospective cohort study (ClinicalTrial.gov number: NCT03023293). At baseline, we recruited pregnant women (20–28 weeks' gestation) aged 20–45 years in a Maternal and Child Health Hospital in Guangzhou, China from March 2017 to November 2018. Individuals diagnosed with preexisting cardiovascular disease, thyroid disease, diabetes mellitus, hematological disease, polycystic ovary syndrome, or mental disorder before conception and those with pregnancy infection or multiple pregnancies were excluded. The cohort was followed at 6 months after delivery.

A total of 789 mother-infant pairs were enrolled. We further excluded those whose dietary data was incomplete (*n* = 35), or protein contributions from each food group were 5 standard deviations below or above the mean protein contributions (*n* = 41). Thus, a total of 713 mother-infant pairs were included in the final analysis. Ethical approval for this cohort was given by the Ethics Committee of the School of Public Health of Sun Yat-Sen University. Written informed consent was provided by all participants.

### Assessment of Maternal Dietary

Maternal dietary information was collected via a validated food frequency questionnaire (FFQ) at baseline survey in the face-to-face interview. FFQ was often used in large-scale population-based dietary surveys and reported to be a useful method to determine maternal food intake during pregnancy (29). The FFQ consisted of 81 food items, covering the most common foods consumed in China, and had been shown to be valid and reproducible among Chinese women in Guangzhou (30). Participants were asked to recall their habitual diet in the past month, including their frequency of consumption (number of times per month, week, or day for each food item) and food portion sizes, and the field staff well-trained filled out the FFQ. The food intake portion per frequency was presented in grams (e.g., 100 g of cooked fish), natural units (e.g., 1 egg), or household measures (e.g., 1 spoon). To help participants quantify their food intake, food photo booklet with standard portion sizes was also provided.

The 2004 Chinese Food Composition Table (31) was applied to convert each food consumption into daily nutrients intake. Maternal daily intake of protein was adjusted for total energy intake using the regression residual method (32). According to the 2016 Chinese Dietary Guidelines (33), 81 food items were aggregated into 10 pre-defined food groups. These groups included cereals and tubers (including grains, mixed beans, and tubers), vegetables, fruits, red meat, poultry, fish and seafood, eggs, dairy and its products, soybeans and its products, and nuts and seeds. Individual daily protein intake from each food group was calculated. Then the percentage of total dietary protein for each food group was calculated for subsequent K-means cluster analysis to determine dietary protein patterns.

### Assessment of Infant Eczema

The information of infant eczema was collected by telephone interview when the infants were 6 months of age. The mothers of infants completed telephone interviews. Eczema was assessed by the symptom questionnaire of eczema, and infant eczema was defined according to the diagnostic criteria in practical pediatrics of Zhu Futang (34). All the telephone interviewers had medical research background and were trained in eczema diagnosis.

### Assessment of Covariates

At baseline survey, the demographics and lifestyle factors during pregnancy were investigated by the face-to-face interview. The information included maternal age, gestational age, parity, educational level, monthly household income per capita, smoking, and alcohol consumption. Smoking status and alcohol use during pregnancy were divided into yes or no. Height (nearest 0.1 cm) was measured by trained clinical nurses, and pre-pregnancy body weight was self-reported. Pre-pregnancy body mass index (BMI, kg/m^2^) was calculated as pre-pregnancy body weight (kg) divided by height squared (m^2^).

Infant birth information was abstracted from hospital records, including infant sex, birth weight, and date of birth. Feeding patterns, breastfeeding duration, solids introduction in 6 months, maternal history of food allergy, family history of allergy diseases (at least one of the immediate family members had eczema, food allergy, drug allergy, asthma, allergic rhinitis, or urticaria), and family history of eczema were collected at 6 months of age by telephone interviews.

### Statistical Analysis

Characteristics of the study population and maternal dietary intakes of each food group by infant eczema were described using proportions for categorical variables or means and standard deviations for continuous variables, with differences tested using *t*-test and chi-square test. And multiple linear regression model was conducted to adjust for potential confounding factors, including maternal delivery age, pre-pregnancy BMI, monthly household income, educational level, maternal history of food allergy, family history of allergy diseases, family history of eczema, gestational age, parity, smoking during pregnancy, and alcohol use during pregnancy.

Dietary protein patterns were derived by K-means cluster analysis. This method was widely performed to analyze dietary patterns (35–37). Participants were categorized into mutually exclusive dietary pattern groups based on protein consumptions of each food group by cluster analysis. Cluster analysis was proved to have reasonable reproducibility and validity (38), and the K-means method has higher repeatability (39). Firstly, the number of clusters (k) was specified. In the present study, cluster analysis was performed varying from 3 to 5 to identify the optimal number of clusters. Then all subjects were assigned to k clusters by calculating Euclidean distances and updating the location of centroids in an iterative process. Finally, the four-cluster was selected because it distinguished meaningful separated clusters, and it presented a reasonable sample distribution in each cluster. These clusters were termed poultry, plant, dairy and eggs, and red meat and fish dietary protein patterns.

Logistic regression models were performed to determine the associations between maternal dietary protein patterns in pregnancy and infant eczema by calculating crude and adjusted odds ratios (ORs) and 95% confidence interval (95% CI). In model 1, we adjusted for maternal delivery age, pre-pregnancy BMI, monthly household income, and educational level. In model 2, we additionally adjusted for maternal history of food allergy, family history of allergy diseases, family history of eczema. In model 3, we further adjusted for gestational age, parity, smoking during pregnancy, alcohol use during pregnancy, daily dietary energy intake. In the last model, infant sex, birth weight, birth season, baby's feeding patterns, breastfeeding duration, and introducing solids in 6 months were further adjusted. To assess confounding arising from other dietary factors during pregnancy, we further adjusted separately for maternal n-3 polyunsaturated fatty acids (n-3 PUFAs), n-6 polyunsaturated fatty acids (n-6 PUFAs), dietary fiber, and Vitamin E intake in sensitivity analysis. All analyses were performed using SAS version 9.4 (SAS Institute Inc., Cary, NC). *P* < 0.05 was considered statistically significant.

## Results

### Characteristics of the Participants

The participant characteristics according to infant eczema status are presented in Table 1. During the first 6 months after birth, 365 out of 713 infants (51.19%) had eczema. Compared to infants without eczema, infants with eczema were more likely to have a family history of allergy diseases (*P* < 0.0001). They also tended to have a positive family history of eczema (*P* = 0.0001). There was a higher proportion of underweight women (BMI < 18.50 kg/m^2^) among mothers of infants with eczema than those without eczema. There was no significant difference between eczema and non-eczema groups among other characteristics.

**Table 1:** Characteristics in a cohort study by categories of eczema^a^.

**Characteristics**	**Total**	**Eczema**	**Normal**	***P***
*N (%)*	713	365 (51.19)	348 (48.81)	
**Baseline characteristics**				
Maternal age, *years*	30.20 ± 4.86	30.13 ± 4.75	30.27 ± 4.98	0.692
Gestational age, *weeks*	25.48 ± 2.35	25.47 ± 2.13	25.49 ± 2.58	0.904
Pre-pregnancy BMI*, kg/m^2^, n (%)*				**0.024**
< 18.50	161 (23.68)	99 (28.05)	62 (18.96)	
18.50–23.99	432 (63.53)	212 (60.06)	220 (67.28)	
23.99–27.99	73 (10.74)	33 (9.35)	40 (12.23)	
≥28.00	14 (2.06)	9 (2.55)	5 (1.53)	
Parity, primiparous*, n (%)*	275 (38.68)	153 (41.92)	122 (35.26)	0.069
Smoking*, n (%)*	38 (5.35)	22 (6.03)	16 (4.64)	0.411
Alcohol use*, n (%)*	25 (3.52)	14 (3.84)	11 (3.18)	0.635
Educational level*, n (%)*				0.082
Junior high school and below	105 (15.11)	42 (11.86)	63 (18.48)	
High or technical secondary school	140 (20.14)	72 (20.34)	68 (19.94)	
Junior college or college	400 (57.55)	216 (61.02)	184 (53.96)	
Postgraduate and above	50 (7.19)	24 (6.78)	26 (7.62)	
Monthly household income*, n (%)*				0.306
< 4,000 RMB	149 (21.56)	69 (19.33)	80 (23.95)	
4,001–6,000 RMB	157 (22.72)	78 (21.85)	79 (23.65)	
6,001–10,000 RMB	176 (25.47)	93 (26.05)	83 (24.85)	
>10,000 RMB	209 (30.25)	117 (32.77)	92 (27.54)	
**Infant's characteristics**				
Sex (boy)*, n (%)*	291 (50.79)	167 (52.85)	124 (48.25)	0.273
Birth weight, *kg*	3.21 ± 0.41	3.22 ± 0.42	3.19 ± 0.40	0.415
Feeding patterns, *n (%)*				0.981
Predominant breastfeeding	307 (43.06)	158 (43.29)	149 (42.82)	
Combined	354 (49.65)	181 (49.59)	173 (49.71)	
Formula feeding	52 (7.29)	26 (7.12)	26 (7.47)	
Breastfeeding duration, *months, n (%)*				0.792
< 4	331 (46.42)	168 (46.03)	163 (46.84)	
4–6	128 (17.95)	69 (18.90)	59 (16.95)	
>6	254 (35.62)	128 (35.07)	126 (36.21)	
Introducing solids in 6 months*, n (%)*	683 (95.79)	350 (95.89)	333 (95.69)	0.894
Maternal history of food allergy*, n (%)*	156 (21.97)	88 (24.18)	68 (19.65)	0.146
Family history of allergy diseases*, n (%)*	351 (49.23)	207 (56.71)	144 (41.38)	** < 0.0001**
Family history of eczema*, n (%)*	52 (7.29)	40 (10.96)	12 (3.45)	**0.0001**

a *Values were presented as mean ± standard deviation, or proportions. The bold values indicated that there were statistical significance (P < 0.05)*.

### Maternal Dietary Intake

Table 2 summarizes the maternal dietary intakes of total energy, animal protein, plant protein, and protein from different food sources, comparing infants with or without eczema. Mothers of infants with eczema consumed more poultry protein sources during pregnancy (*P* = 0.005). No statistical differences were observed in maternal intakes of total energy, animal protein, plant protein or protein from other food sources (cereals and tubers, vegetables, fruits, red meat, fish and seafood, eggs, dairy, soybean, and nuts and seeds) between different eczema status categories.

**Table 2:** Dietary consumption in a cohort study by categories of eczema^a^.

	**Total**	**Eczema**	**Normal**	***P***	**Adjusted *P*^c^**
**Dietary intake^b^**	**(*N* = 713)**	**(*N* = 365)**	**(*N* = 348)**		
Total energy*, Kcal/d*	1789.95 ± 495.55	1817.41 ± 502.27	1761.14 ± 487.45	0.130	0.319
Animal protein*, g/d*	40.83 ± 13.49	41.35 ± 13.66	40.29 ± 13.32	0.296	0.757
Plant protein*, g/d*	30.35 ± 5.52	30.24 ± 5.49	30.46 ± 5.55	0.586	0.490
**Protein source*****, g/d***					
From cereals and tubers	17.96 ± 4.80	18.05 ± 4.91	17.87 ± 4.68	0.626	0.315
From vegetables	4.32 ± 2.19	4.39 ± 2.24	4.24 ± 2.14	0.370	0.198
From fruits	1.74 ± 1.06	1.74 ± 1.13	1.75 ± 0.99	0.935	0.637
From red meat	16.76 ± 9.82	16.70 ± 9.63	16.83 ± 10.03	0.860	0.496
From poultry	4.82 ± 4.14	**5.24** **±** **4.43**	**4.38** **±** **3.77**	**0.005**	**0.005**
From fish and seafood	6.99 ± 6.45	7.27 ± 6.77	6.69 ± 6.10	0.225	0.259
From eggs	4.84 ± 3.21	4.79 ± 3.01	4.89 ± 3.41	0.682	0.436
From dairy	7.45 ± 5.12	7.37 ± 5.51	7.54 ± 4.69	0.664	0.224
From soybean	2.89 ± 2.78	2.77 ± 2.76	3.02 ± 2.79	0.228	0.840
From nuts and seeds	3.30 ± 4.01	3.17 ± 3.86	3.45 ± 4.16	0.358	0.380

a *Values were presented as mean ± standard deviation*.

b *Estimated intake energy adjusted by the residual method*.

c *Multiple linear regression analysis was adjusted for maternal age, pre-pregnancy BMI, monthly household income, educational level, maternal history of food allergy, family history of allergy diseases, family history of eczema, gestational age, parity, smoking during pregnancy, and alcohol use during pregnancy*.

*The bold values indicated that there were statistical significance (P < 0.05)*.

### Maternal Dietary Protein Patterns

**Figure 1:**
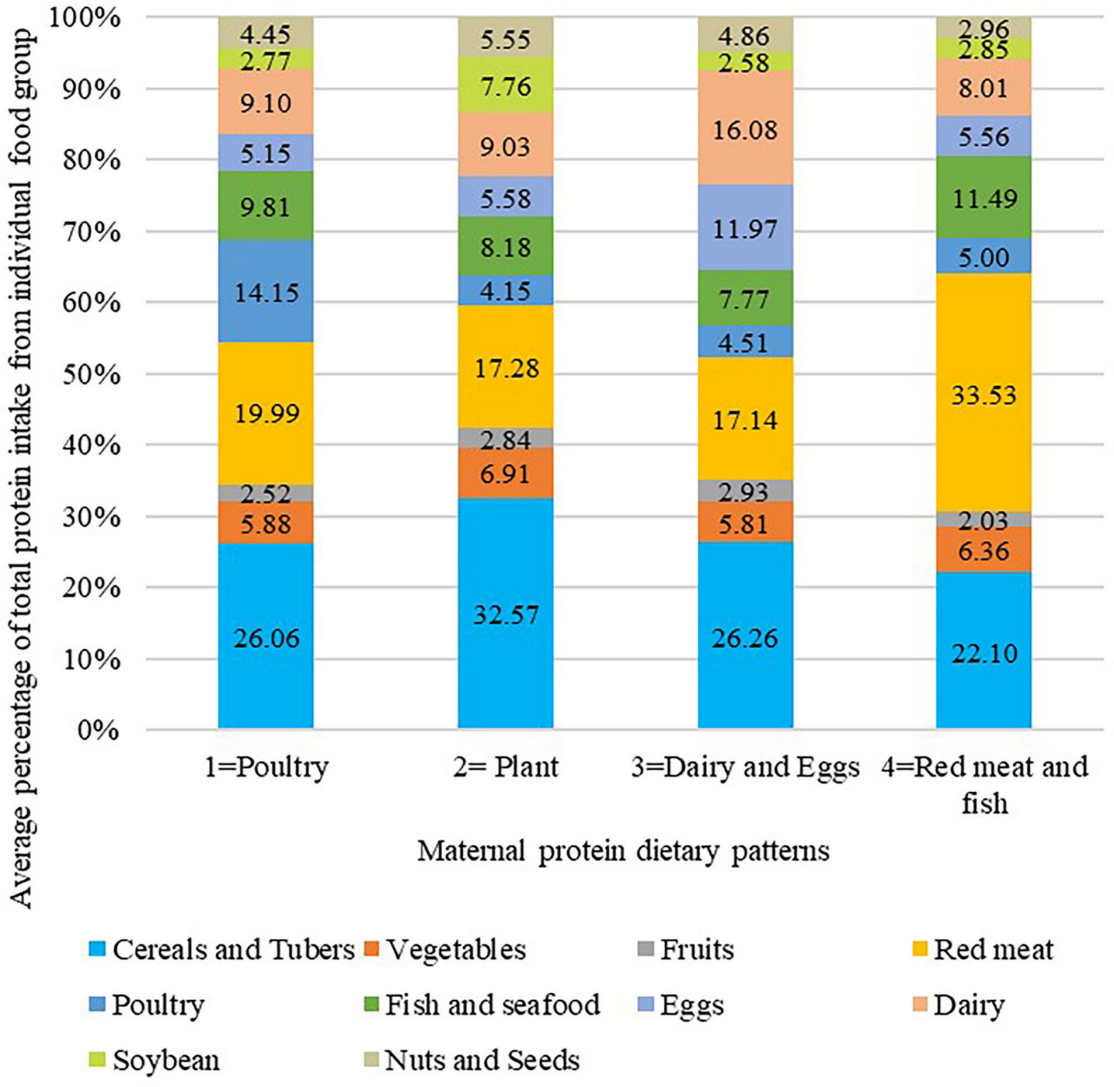
Average percentage of total protein intake from individual food group across protein food cluster analysis. A K-means cluster analysis was used to classify participants into mutually exclusive groups. Naming of clusters was determined by the value which represent the highest consumption of one or two food groups compared with other clusters. The results of Analysis of Variance showed that there were significant differences among the percentage contribution of protein intake from each food group between four dietary pattern groups (*P* < 0.05).

Participants were divided into four different dietary protein pattern groups, named by their predominant protein intake sources. Percentage contribution of protein intake from each food group across four dietary patterns is listed in Figure 1. The results of Analysis of Variance showed that there were significant differences among the percentage contribution of protein intake from each food group between four dietary pattern groups (*P* < 0.05, data not shown). Compared to other groups, women who exhibited poultry dietary protein pattern consumed relatively higher protein from poultry (mean percentage contribution of protein intake from poultry: 14.15%, *n* = 147). The plant dietary protein pattern (*n* = 179) was characterized by a relatively higher protein intake from cereals and tubers (32.57%), vegetables (6.91%), soybean (7.76%), and nuts and seeds (5.55%). The dairy and eggs dietary protein pattern (*n* = 171) presented with higher protein consumption from dairy (16.08%) and eggs (11.97%). The red meat and fish dietary protein pattern (*n* = 216) was characterized by higher protein intake from red meat (33.53%) and fish and seafood (11.49%).

### Maternal Dietary Protein Patterns and the Risk of Infant Eczema

The univariate and multivariate regression analysis for the association between maternal dietary protein pattern during pregnancy and risk of infant eczema were listed in Table 3. The cumulative incidence of infant eczema in the poultry, plant, dairy and eggs, and red meat and fish dietary protein patterns was 61.2, 45.8, 48.0, 51.4%, respectively. After controlling for potential confounders, compared to the poultry dietary protein pattern, the multivariable-adjusted ORs (95% CI) for infant eczema were 0.572 (0.330–0.992) and 0.478 (0.274–0.837) in the plant pattern and dairy and eggs pattern, respectively. For the red meat and fish dietary protein pattern, the association was not statistically significant.

**Table 3:** Association between maternal dietary protein patterns and infant eczema.

	**Dietary Patterns**			
**Model**	**Poultry**	**Plant**	**Dairy and Eggs**	**Red meat and Fish**
	**(*N* = 147)**	**(*N* = 179)**	**(*N* = 171)**	**(*N* = 216)**
Cases (Incidence^a^)	90 (61.2%)	82 (45.8%)	82 (48.0%)	111 (51.4%)
Unadjusted ORs (95% CI)	1.00	**0.535 (0.344–0.834)**	**0.584 (0.373–0.913)**	0.670 (0.437–1.025)
Adjusted ORs (95% CI)^b^
Model 1	1.00	**0.563 (0.350–0.904)**	**0.503 (0.310–0.816)**	**0.581 (0.368–0.919)**
Model 2	1.00	**0.574 (0.355–0.927)**	**0.491 (0.301–0.803)**	**0.561 (0.353–0.893)**
Model 3	1.00	**0.579 (0.354–0.947)**	**0.479 (0.290–0.790)**	**0.578 (0.360–0.928)**
Model 4	1.00	**0.572 (0.330–0.992)**	**0.478 (0.274–0.837)**	0.654 (0.388–1.103)

a *The cumulative incidence of infant eczema in four dietary protein patterns*.

b *>Model 1 was adjusted for maternal age, pre-pregnancy BMI, monthly household income and educational level*.

*Model 2 was further adjusted for maternal history of food allergy, family history of allergy diseases, family history of eczema*.

*Model 3 was further adjusted for gestational age, parity, smoking during pregnancy, alcohol use during pregnancy, daily dietary energy intake*.

*Model 4 was further adjusted for infant sex, birth weight, birth season, baby's feeding patterns, breastfeeding duration, and introducing solids in 6 months.*

*The bold values indicated that there were statistical significance (P < 0.05)*.

### Sensitivity Analysis

In a sensitivity analysis, compared to the red meat and fish dietary protein pattern, no association was observed between other dietary protein patterns and risk of infant eczema after adjusting for potential confounders (see Supplementary Table 1). Additional separate adjustment for maternal intake of n-3 PUFAs, n-6 PUFAs, dietary fiber, and Vitamin E during pregnancy did not substantially alter the main findings (see Supplementary Table 2).

## Discussion

In this prospective mother-infant cohort study, maternal consumption of poultry protein during pregnancy was higher in the eczema group than the control group. Four dietary protein patterns among pregnant women were identified, labeled the poultry, plant, dairy and eggs, and red meat and fish dietary protein patterns. Compared with the poultry dietary pattern, the plant pattern, and dairy and eggs pattern were associated with reduced risk of infant eczema at 6 months of age. This is the first prospective study to investigate the association between maternal dietary protein patterns during pregnancy and infant eczema, and this study provides epidemiological evidence to support the associations between maternal diet and offspring allergic diseases.

Evaluating the association between maternal dietary protein and infant eczema from a whole-diet perspective is necessary. Antigens composition and amino acid profiles vary in different dietary protein patterns. The mechanisms of divergent associations between maternal dietary intake and infant eczema remain unclear. Maternal dietary protein patterns may influence infant eczema through several pathways below. Firstly, there is growing evidence that maternal microbiota influences allergic diseases in offspring (40, 41). Animal experiments have shown that the composition and function of microbiota, especially in the gastrointestinal tract, can be modulated by dietary proteins (42, 43), including protein sources diversity and protein level (44). Recent studies have indicated that maternal gut microbiota could be transferred to the fetus (45, 46), and microbes are detected in human placenta and amniotic fluid (47). Prenatal microbial exposures are necessary to establish and develop a healthy nascent microbiome (48), which may have major impacts on immune system maturation (49). Secondly, epigenetic mechanisms provide a new explanation for the development of allergic diseases (50–52). Amino acids play an important role in the epigenetic mechanisms, especially DNA methylation. Some amino acids (such as methionine, serine), as dietary methyl donors, can alter epigenetic characteristics (53, 54). And amino acids profile and transport capacity from mother to fetus are influenced by the composition and content of maternal dietary proteins (55). Immune development *in utero*, including T-helper type 1 (Th)1 and Th2 differentiation patterns, regulatory T cell differentiation, and Th17 development, is under epigenetic control, indicating marked plasticity in early T cell differentiation (50).

In our study, the poultry dietary protein pattern had the highest incidence of infant eczema. We also found that mothers of infants with eczema consumed more protein from poultry food source during pregnancy than mothers of infants without eczema. This result was in line with a previous study in Australia demonstrating that maternal pre-conception poultry intake was positively associated with offspring eczema (56). In the adult literature, two studies also found that diets with high intake of poultry were associated with allergic diseases (57, 58). Because of the potential adverse association between poultry protein intake and eczema, the poultry dietary protein pattern, consisting of more poultry protein, was used as a benchmark for comparison.

We did not find significant difference between the red meat and fish pattern and the poultry dietary pattern on risk of infant eczema. Participants in the red meat and fish dietary pattern consumed a relatively higher protein intake from red meat and fish (even more than 1/3 of protein intake from red meat). In a previous prospective study in Japan, higher maternal meat intake during pregnancy was independently related to an increased risk of infant eczema, whereas no relationship was observed between maternal intake of fish and the risk of eczema (59). However, the effect of maternal fish intake on offspring allergic diseases remains controversial. A pooled analysis of 18 birth cohorts found that maternal fish and seafood intake during pregnancy was not associated with offspring wheeze, asthma, and allergic rhinitis (60). Similarly, a meta-analysis indicated that fish intake during pregnancy had no effect on the risk of eczema (61). However, several epidemiological studies showed protective effects from fish consumption in pregnancy on childhood allergic diseases (62–64). Fish, especially oily fish, is the main dietary source of n-3 PUFAs, which have been suggested to have anti-inflammatory properties and may decrease the risk of allergic diseases (65). In sensitivity analysis of the current study, compared to the red meat and fish pattern, other dietary protein patterns were not associated with a reduced risk of infant eczema, that might be due to the interaction of various food ingredients in this dietary pattern.

Compared to the poultry dietary protein pattern, the plant pattern, characterized by higher protein intake from cereals and tubers, vegetables, soybean, and nuts and seeds, was associated with a reduced risk of infant eczema. And such association was observed in the dairy and eggs pattern, which was characterized by higher protein intake from dairy and eggs. A previous ecological study based on the International Study of Asthma and Allergies in Childhood (ISAAC) reported an inverse relationship between protein from cereals, nuts, and vegetables and childhood eczema (66). A cross-sectional study in Japan demonstrated that higher intake of soy protein was associated with a reduced prevalence of allergic rhinitis among pregnant women (67). In addition, our results were consistent with those of previous studies that indicated a protective effect of maternal intake of vegetables, fruits, and dairy products on infant eczema (19, 68, 69). These foods were rich in antioxidant (such as vitamin C and β-carotene), vitamin D and calcium, which might have potential protective effects on eczema (69, 70). Furthermore, it is well-known that immune system development begins *in utero*, and immune dysfunction in children who have allergic diseases has been manifest at birth (10). Evidence is accumulating that exposure to food allergens [commonly present in milk, fruit, eggs, nuts, wheat, and fish (21)] during pregnancy may reduce the risk of allergic diseases in offspring (18, 19, 68, 69, 71). And encounter with food allergens *in utero* via maternal dietary could induce immune tolerance rather than sensitization (10, 18), which may explain that the plant and dairy and eggs dietary protein patterns are associated with a reduced risk of infant eczema compared with the poultry dietary protein pattern.

There are several limitations that should be considered in our study. Firstly, we measured maternal dietary intake via FFQ, which might lead to recall bias and imprecise quantification of dietary intake (29). However, reliability and validity of FFQ among Chinese women have been validated to be good overall (30). And visual aids such as food photographs of portion sizes were provided to help them quantify their food intake. Secondly, data of infant eczema was based on parent-reported by telephone interview, which might lead to misdiagnose and misclassification. However, this misclassification of the outcome was likely to be non-differential in four dietary protein patterns, and the bias effect estimates was expected toward the null. And well-trained investigators can help mothers determine whether their offspring have ever had eczema. Thirdly, although we adjusted numerous potential confounders, residual confounding may also influence the development of infant eczema. Finally, it remains to be seen whether associations between maternal dietary protein patterns during pregnancy and infant eczema persists beyond the age of 6 months. Therefore, continued follow-up of our cohort is required to observe the long-term effects of maternal diet during pregnancy on childhood eczema.

## Conclusion

The current prospective cohort study suggested that maternal dietary protein during pregnancy may influence the development of infant eczema at the age of 6 months. Compared with the poultry dietary protein pattern, the maternal plant pattern and dairy and eggs dietary protein pattern were associated with a reduced risk of infant eczema. Optimizing the composition of proteins from different food sources during pregnancy may be a promising strategy for reducing the risk of infant eczema, and further researches regarding biological mechanisms and interventional trials are necessary to confirm our findings.

## Data Availability Statement

The datasets analyzed in this article are not publicly available. Requests to access the datasets should be directed to LC, caili5@mail.sysu.edu.cn.

## Ethics Statement

The studies involving human participants were reviewed and approved by the Ethics Committee of the School of Public Health of Sun Yat-Sen University. Written informed consent to participate in this study was provided by the participants' legal guardian/next of kin.

## Author Contributions

LC, JJ, WW, and JZ conceived the study. JZ and WW extracted data and performed all statistical analyses. JZ prepared the manuscript draft. LC, JJ, and WW revised the initial manuscript. LC, NT, and WW critically edited language. LC and JJ supervised the study. LC obtained funding and material support. JZ, WW, NT, LC, JJ, and YC performed the investigation. All authors critically revised drafts of the manuscript and approved the final manuscript.

## Conflict of Interest

The authors declare that the research was conducted in the absence of any commercial or financial relationships that could be construed as a potential conflict of interest.
