# Supplementary material for: Maternal Dietary Protein Patterns During Pregnancy and the Risk of Infant Eczema: A Cohort Study
Source: Front Nutr. 2021 Jun 2;8:608972. doi: 10.3389/fnut.2021.608972 (PMC8206490; doi:10.3389/fnut.2021.608972)
Supplement: Supplementary file 1 [file Table_1.pdf]

Supplementary Materials

TABLE 1 Association between maternal dietary protein patterns and infant eczema

Model	Dietary Patterns			
	Red meat and Fish (*N*=216)	Plant (*N*=179)	Dairy and Eggs (*N*=171)	Poultry (*N*=147)
Cases (Incidence[1])	111 (51.4%)	82 (45.8%)	82 (48.0%)	90 (61.2%)
Unadjusted ORs (95% CI)	1.00	0.800 (0.538-1.189)	0.872 (0.583-1.302)	1.494 (0.976-2.286)
Adjusted ORs (95% CI)[2]				
Model 1	1.00	0.968 (0.636-1.473)	0.865(0.564-1.326)	**1.720 (1.089-2.716)**
Model 2	1.00	1.022 (0.666-1.568)	0.875 (0.566-1.353)	**1.781 (1.120-2.832)**
Model 3	1.00	1.001 (0.646-1.553)	0.828 (0.531-1.292)	**1.730 (1.078-2.778)**
Model 4	1.00	0.874 (0.536-1.426)	0.731 (0.444-1.206)	1.529 (0.907-2.577)

[1] The cumulative incidence of infant eczema in four dietary protein patterns.

[2] Model 1 was adjusted for maternal age, pre-pregnancy BMI, monthly household income and educational level;
Model 2 was further adjusted for maternal history of food allergy, family history of allergy diseases, family history of eczema;
Model 3 was further adjusted for gestational age, parity, smoking during pregnancy, alcohol use during pregnancy, daily dietary energy intake;
Model 4 was further adjusted for infant sex, birth weight, birth season, baby's feeding patterns, breastfeeding duration, and introducing solids in 6 months.
