# Supplementary material for: Maternal Dietary Protein Patterns During Pregnancy and the Risk of Infant Eczema: A Cohort Study
Source: Front Nutr. 2021 Jun 2;8:608972. doi: 10.3389/fnut.2021.608972 (PMC8206490; doi:10.3389/fnut.2021.608972)
Supplement: Supplementary file 2 [file Table_2.pdf]

Supplementary Materials

TABLE 2 Association between maternal dietary protein patterns and infant eczema

	Dietary Patterns			
	Poultry (*N*=147)	Plant (*N*=179)	Dairy and Eggs (*N*=171)	Red meat and Fish (*N*=216)
Model 1	1.00	**0.565 (0.322-0.993)**	**0.450 (0.254-0.796)**	0.639 (0.375-1.088)
Model 2	1.00	**0.569 (0.325-0.998)**	**0.459 (0.259-0.811)**	0.631 (0.371-1.072)
Model 3	1.00	**0.547 (0.310-0.965)**	**0.449 (0.254-0.795)**	0.635 (0.373-1.079)
Model 4	1.00	**0.542 (0.306-0.959)**	**0.445 (0.251-0.789)**	0.625 (0.368-1.063)

Logistic regression model was adjusted for maternal age, pre-pregnancy BMI, monthly household income and educational level, maternal history of food allergy, family history of allergy diseases, family history of eczema, gestational age, parity, smoking during pregnancy, alcohol use during pregnancy, daily dietary energy intake, infant sex, birth weight, birth season, baby's feeding patterns, breastfeeding duration, and introducing solids in 6 months;
Model 1 was further adjusted for maternal intake of n-3 polyunsaturated fatty acids (n-3 PUFAs) during pregnancy;
Model 2 was further adjusted for maternal intake of n-6 polyunsaturated fatty acids (n-6 PUFAs) during pregnancy;
Model 3 was further adjusted for maternal intake of dietary fiber during pregnancy;
Model 4 was further adjusted for maternal intake of Vitamin E during pregnancy.
